# Fabrication of Screen Printing-Based AgNWs Flexible Transparent Conductive Film with High Stability

**DOI:** 10.3390/mi11121027

**Published:** 2020-11-24

**Authors:** Jianjun Yang, Wei Zeng, Yaxin Li, Zichuan Yi, Guofu Zhou

**Affiliations:** 1 College of Electron and Information, University of Electronic Science and Technology of China, Zhongshan Institute, Zhongshan 528402, China; yizichuan@163.com; 2 School of Optoelectronic Science and Engineering, University of Electronic Science and Technology of China, Chengdu 610054, China; pttcrispy@gmail.com (W.Z.); leiyafangzhou@163.com (Y.L.); 3 South China Academy of Advanced Optoelectronics, South China Normal University, Guangzhou 510006, China; guofu.zhou@m.scnu.edu.cn

**Keywords:** flexible transparent conductive films, silver nanowires, screen printing

## Abstract

Flexible transparent conductive thin films (TCFs) prepared from Silver nanowires (AgNWs) have attractive features of low cost, flexibility, and solution-processed, but the usual manufacturing methods could still be hard to be scaled up. In addition, large-scale/large-area fabrication process with industrialized potential is strongly needed. In this paper, the flexible TCFs with high stability are obtained via using screen printing method to print the AgNWs inks on a flexible and transparent substrate. The micro-structure of the AgNWs patterns is investigated by optical microscope and scanning electron microscope. Furthermore, the sheet resistance, light transmittance, and film thickness of the AgNWs patterns prepared under different conditions are characterized to explore the influence of different factors on its optical and electrical properties.

## 1. Introduction

As next generation optoelectronic devices tend towards mobile and wearable devices, flexible electronics became of fundamental importance in the development of efficient and stretchable optoelectronic devices such as organic light-emitting diodes (OLEDs), touch screens, e-readers, electronic papers, and organic photovoltaics (OPVs) [1,2,3,4,5]. An essential component behind all of these devices and systems is transparent conductive film, which is a thin film material with high light transmittance and excellent electrical conductivity in the visible light range. Indium tin oxide (ITO) is currently the material primarily selected for the production of TCFs. However, the expensive and fragile ITO will easily produce cracks under repeated bending. Meanwhile, the limited reserves of the raw materials and the technique normally employed in the fabrication of ITO film result in increased cost, which hinders the development of the low-cost/large-area fabrication. In terms of OPVs, for instance, the use of ITO as TCFs has accounted for >50% of the total material and process cost [6,7]. Therefore, it is crucial to seek alternative materials for TCFs to replace ITO.

Several emerging materials such as graphene, carbon nanotubes (CNTs), and conductive polymers had been explored as the next-generation TCF materials to substitute ITO [8]. However, there were vital issues of applying them as TCFs. For instance, the graphene has difficulties in mass production and there are fundamental concerns of its horizontal conduction in large area. The large contact resistance at the junction of CNTs should be treated as a major difficulty in urgent need of solution. Simultaneously, the long-term stability of conductive polymers (e.g., PEDOT:PSS) also needs to addressed. The metal TCFs, including the metal nanowires, metal grid, and ultrathin metal film, could be manufactured with excellent optical and electrical properties, which were of great promise to replace ITO. Among them, silver nanowires were considered to be the best potential alternative to ITO in the next generation of flexible TCFs due to their excellent electrical and thermal conductivity, good mechanical stability, acceptable price, and its oxide still has a certain conductivity [9,10,11,12].

To achieve the goal of large-scale construction of stretchable/wearable flexible electronic products, the major challenge is not only to develop the manufacturing technology of low-cost flexible TCFs, but the development of a solution-phase coating process is still urgently needed for the realization of low-cost flexible electronics in mass production [9]. Printed electronics are a significant enabling technology for the development of low-cost, large-area, and flexible optoelectronic devices [13,14,15,16], and these have drawn tremendous interest during the past few decades [17,18,19,20]. Large-scale fabrication could be efficiently enabled by means of diverse printing techniques such as screen printing, inkjet printing, and gravure printing [21,22]. In existing printing methods, screen printing was the most widely used technique due to its low capital cost, simple operation, versatile pattern designs, and little waste, which uses emulsion screens, mesh-mount, or frameless metal stencils to deposit inks and pastes onto various substrates [9,17,18,19,23,24].

Hemmati et al., investigated the rheological behavior of the AgNWs suspension, and the ink was screen-printed onto flexible polycarbonate substrate [25]. However, the conductivity of the printed patterns was not reported, and the quality and resolution of the printed patterns were low. Zhou and co-workers reported the first fully screen-printed topgated thin-film transistors (TFTs) array on flexible substrates using silver-based conductive inks for source, drain, and gate electrodes [9]. Despite great success being realized in the field of flexible screen-printed electronics, few have successfully developed screen-printable inks with high stability and conductivity for wearable electronic applications [26,27,28]. Therefore, many eventful challenges still remain in the development of high-quality screen printable inks for TCFs.

In this paper, the fabrication of conductive printed patterns of AgNWs on flexible poly(ethylene terephthalate) (PET) via screen-printing was reported. Silver nanowires were prepared by the polyol solvothermal method, and the silver nanowires conductive inks developed by a specific formulation were used to gain a uniform film with high stability by screen printing [29,30]. The factors of preparation were demonstrated including ink formulation, substrate treatment, patterning, and postprocessing. The effects of the amount of silver nanowires, heat treatment temperature, and bending times on the microstructure, optical and electrical properties of samples were investigated.

## 2. Materials and Methods

### 2.1. Materials

Poly(vinylprrolidone) (PVP, K30, Mw ≈ 10,000), was purchased from Hefei Vigon Materials Technology Co., Ltd. Silver nitrate (≥99.8%) was purchased from Shanghai Aladdin Biochemical Technology Co., Ltd. Ferric chloride hexahydrate (AR) was purchased from Shanghai Meiruil Chemical Technology Co., Ltd. Ethylene glycol (AR) and ethanol absolute (99.7%) were purchased from Tianjin Damao Chemical Co., Ltd. (Hydroxypropyl) methyl cellulose (average Mn ≈ 10,000) was obtained from ChongYao Biological Co., Ltd. Fluorocarbon surfactant Zonyl FS-3100 was purchased from Vtolo Chemical Co., Ltd. Defoamer DF-105 was requested from DOW Company. Silver nanowires were synthesized in our laboratory. The average diameter and length of the AgNWs were 25–35 nm and 25–40 μm. All the chemicals were used as received.

### 2.2. Methods

#### 2.2.1. Pretreatment of PET Substrate

PET is one of the most commonly used flexible substrate materials with extremely high light transmittance. However, the surface of PET is hydrophobic, which is not conducive to the adhesion of silver nanowires. Therefore, the surface of PET needs to be pretreated to improve its hydrophilicity. Firstly, piranha solution (A mixture of concentrated sulfuric acid and 30% hydrogen peroxide in 7:3) was prepared and placed in fume hood to cool to room temperature. Then cut the PET into 300 mm × 300 mm, tear off the protective film and float it on the surface of the piranha solution. Finally, remove the PET after 2 h, rinse the surface with deionized water several times, and then dry it with nitrogen.

#### 2.2.2. Preparation of AgNWs Inks

AgNWs were prepared by polyol solvothermal method. First, the distilled water, Ethylene glycol (AR), (hydroxypropyl)methyl cellulose, Zonyl FS-3100, and DF-105 were mixed at the weight ratio of 7:6:1:0.005:0.02, and sonicated for 2 h to make the additive solution. Then, AgNWs solution (1 wt% in ethanol absolute), distilled water, and the additive solution were mixed at the weight ratio of 4:4:1 using magnetic stirrer at 1000 rpm to agitate for 1 h, followed by drying the dispersion completely via vacuum drying. Different amounts of distilled water were added back to the dried mixture to prepare different proportions of ink. Ultimately, the mixtures were agitated by using a magnetic stirrer at 1000 rpm for 1 h to obtain the final AgNWs inks.

#### 2.2.3. Screen Printing and Post Treatment

The Screen Printer (RH-2030P) was purchased from Dongguan and Silk Screen-printing Equipment Factory, and a 300 mm × 300 mm precision screen mesh (400 mesh count) ordered from Zhengda Siyin Co., Ltd. was used. The screen mesh placed on PET was scraped at an angle approximately equal to 45° and a constant speed of 50mm·s^−1^ by a squeegee with ergonomic holder, and the ink would be printed on the PET under the action of shearing force. The printed patterns were annealed to evaporate the solvent to obtain the final conductive AgNWs patterns.

The microstructures of the samples were taken on Olympus (BX-51M) optical microscope and ZEISS (EVO-10) scanning electron microscopy (SEM). The transmittance of the samples was measured by using a transmittance meter GZ502A (Shanghai Guangzhao Photoelectric Technology Co., Ltd., Shanghai, China). The sheet resistance was tested through using four-point probes SX-1944 (Suzhou Baishen Technology Co., Ltd., Suzhou, China). The thickness of the printed pattern film was gauged via stylus profiler Dektak -XT (Shanghai Boyue Intruments Co., Ltd., Shanghai, China).

## 3. Results and Discussion

**Figure 1:**
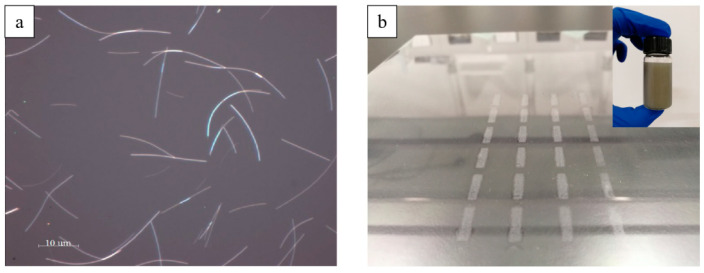
(**a**) Optical microscope image of prepared silver nanowires. (**b**) The photo of screen-printed AgNWs patterns; the inserted image was as-synthesized silver nanowires ink.

It could be found from Figure 1a that the length of the prepared silver nanowires was about 30 μm. Figure 1b showed the screen-printed AgNWs patterns, and the photo of as-synthesized silver nanowires ink was inserted.

**Figure 2:**
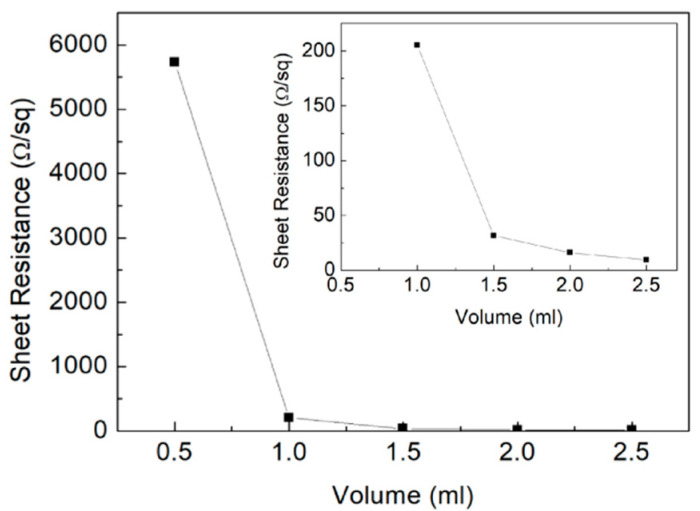
The relationship between sheet resistance of the printed AgNWs patterns and amount of silver nanowires ink. The inserted was a local enlarged image and photo of prepared silver nanowires ink.

The relationship between sheet resistance of the printed AgNWs patterns and amount of silver nanowires ink is shown in Figure 2. The amounts of silver nanowires ink in Figure 2 were 0.5, 1.0, 1.5, 2.0, and 2.5 mL, and the treated temperature of the samples was 120 °C. As in Figure 2, when the amount of silver nanowries ink was 0.5 mL, the sheet resistance of the printed AgNWs patterns was 5730 Ω·sq^−1^. If the amount of silver nanowries ink was 1.0 mL, the sheet resistance dropped sharply to 205.1 Ω·sq^−1^. If the amount of silver nanowries ink was 1.5 mL, the sheet resistance of the printed AgNWs patterns decreased to 31.68 Ω·sq^−1^. When the amount of silver nanowires ink was 2.0 mL, the sheet resistance of the printed AgNWs patterns was 16.12 Ω·sq^−1^. If the amount of silver nanowires ink was 2.5 mL, the sheet resistance of the printed AgNWs patterns was 9.57 Ω·sq^−1^. Therefore, we recommend using at least 2.0 mL of ink.

It was obvious that the conductive behavior of the silver nanowires ink conforms to the infiltration model. After surpassing a certain level of conductive ink loading, the electrical performance was slightly improved. Due to the increase in the amount of conductive ink, more particles filled the gaps among the silver nanowires grid. Meanwhile, more silver nanowires interspersed the substrate through the screen to cause a rapid increasing number of conductive paths formed. Consequently, the constitution of the denser silver nanowires conductive network led to surface resistivity of the printed AgNWs patterns, as the conductive pattern decreases as the amount of ink increases. The percolation model illustrated the relationship between the weight of the added conductive material and the resistivity achieved.

**Figure 3:**
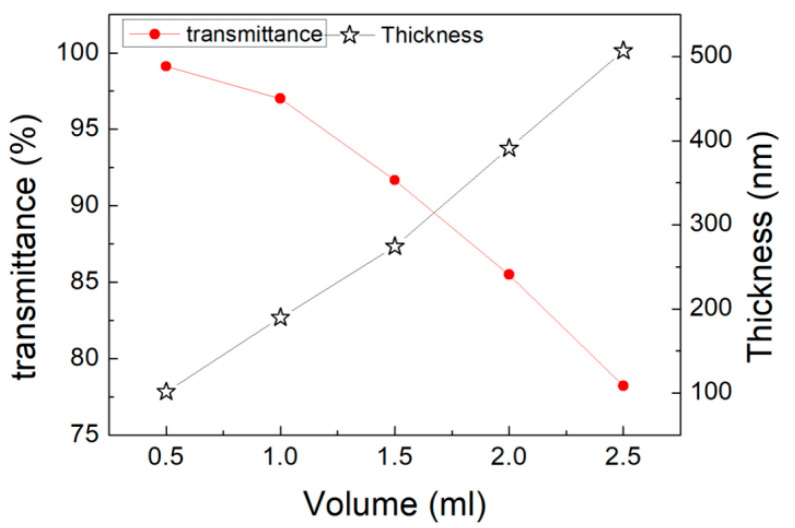
The relationship among transmittance, thickness and amount of silver nanowires ink.

With the growing amount of ink volume as in Figure 3, the film thickness of conductive patterns increased while the light transmittance of the film decreased. Evidently, the accumulation of more silver nanowires and nanoparticles would result in the thickening of the film. The transmittance of the patterns depended on the aspect ratio of the conductive materials, the density, the material size, and the chemical structure. Compared with other factors, it was crucial that the amount of ink affected the density of the conductive network, which is a significant argument for the decrease in transmittance of the thin film.

**Figure 4:**
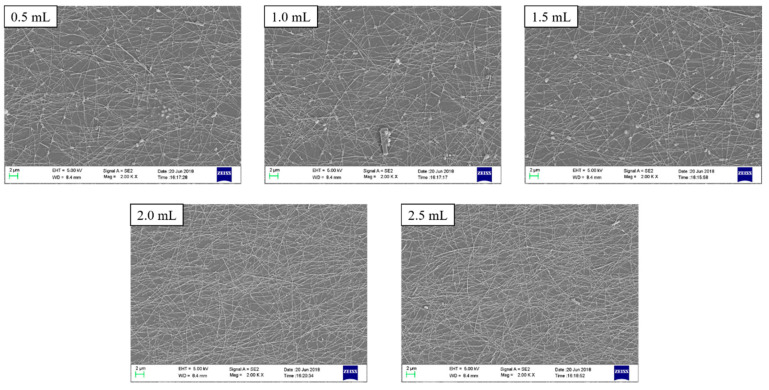
SEM images of the printed AgNWs patterns with different amounts of silver nanowires ink.

The SEM images of the samples are shown in Figure 4. It could be found that, when the amount of silver nanowires was 0.5 mL, only a small amount of silver nanowires covered the surface of the PET; the loose conductive pattern and the sparse conductive network caused the sheet resistance to be large. As the amount of silver nanowire conductive ink increased, more silver nanowires were deposited on the PET surface to form more conductive pathways. It was believed that the conductive contact between the silver nanowires enhanced as the amount of silver nanowires increased.

**Figure 5:**
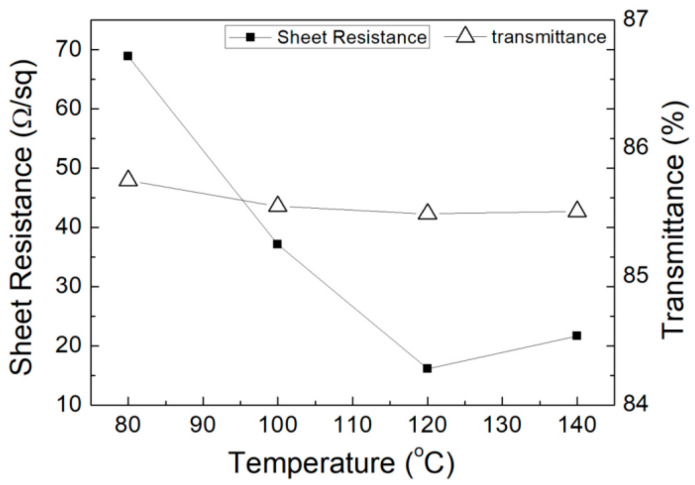
Relationship among the sheet resistance of the printed AgNWs patterns, transmittance and treatment temperature.

The relationship between the sheet resistance of the printed AgNWs patterns (2.0 mL silver nanowires ink) and treated temperature is shown in Figure 5. It is apparent that when the treatment temperature was raised from 80 °C to 120 °C, the sheet resistance decreased remarkably. After that, the sheet resistance of the conductive patterns slightly raised as the processing temperature increased, and the sheet resistance at the lowest point reached 16.12 Ω·sq^−1^ at 120 °C These results demonstrated that there were some differences in the state of the permeable network composed of conductive materials, depending on the treatment temperature. The contact resistance at the interface among the silver nanowires was considered to be extremely affected by the compressive contact stress caused by the thermal effect. In addition, as the solvent evaporated, due to the different volatilization temperatures of solvents, such as surfactants, defoamers, the AgNWs gradually gathered together to form a continuous conductive path before the temperature reached 120 °C. The conductivity was enhanced in this way. However, when the temperature exceeded 120 °C, the sparseness of the conductive network due to the drastic reduction in solubility of methyl cellulose could result in a slight increase in the sheet resistance.

**Figure 6:**
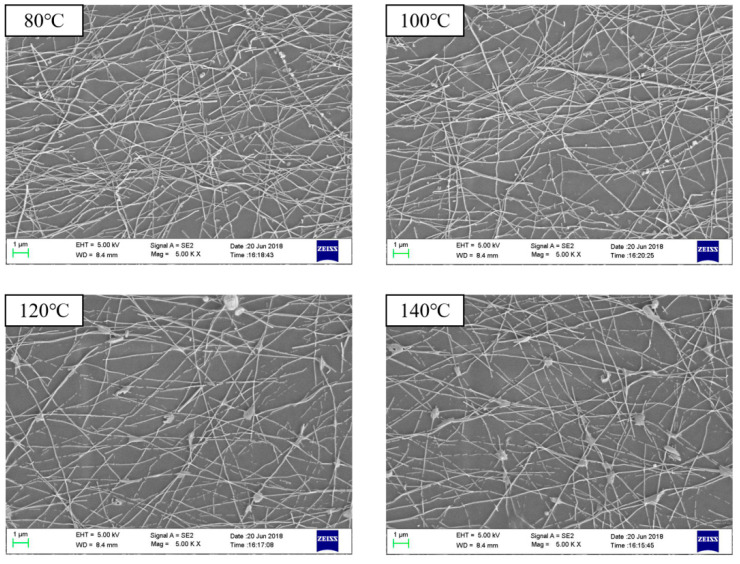
SEM images of the AgNWs patterns at different treatment temperatures.

The SEM images of the printed AgNWs patterns (2.0 mL silver nanowires ink) at different treated temperatures is shown in Figure 6.

**Figure 7:**
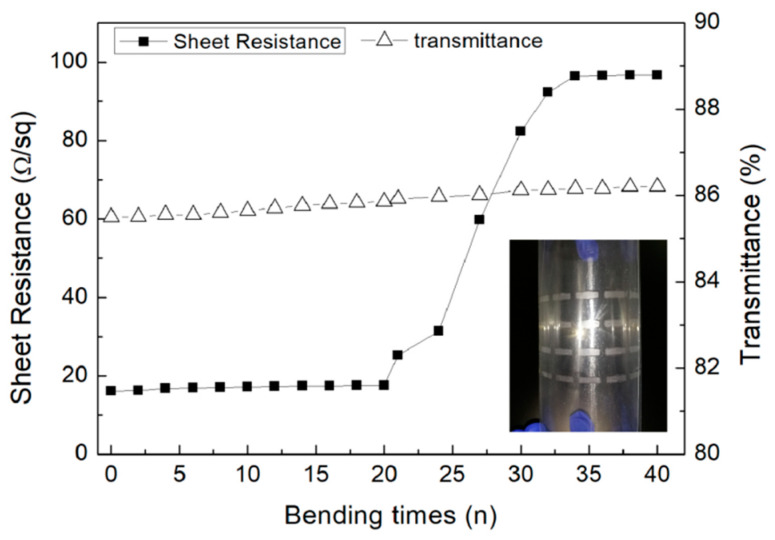
The relationship among the sheet resistance of the printed AgNWs patterns and transmittance with different bending cycles. The inserted photo was the way to bend the printed patterns.

In order to investigate the stability of the flexible TCFs after severe deformation, the PET was bent for several bending cycles in the manner as shown in the inserted image of Figure 7; this bending method allowed the printed patterns to deform considerably. The sheet resistance and transmittance of the printed AgNWs patterns after the number of bending cycles were measured, which was conducive to explore the electrical and optical properties of the thin film in the condition of rapid deformation. Figure 7 illustrates the changes of sheet resistance and transmittance of the flexible TCFs after different bending cycles. There was an inconspicuous rate of change in sheet resistance when the number of bending cycles was less than 20 times. However, when the bending cycles reached 21, the sheet resistance changed greatly, and the changes became more severe with the increasing number of bending cycles. When the number of bending cycles exceeded 40 cycles, the sheet resistance remained almost unchanged.

**Figure 8:**
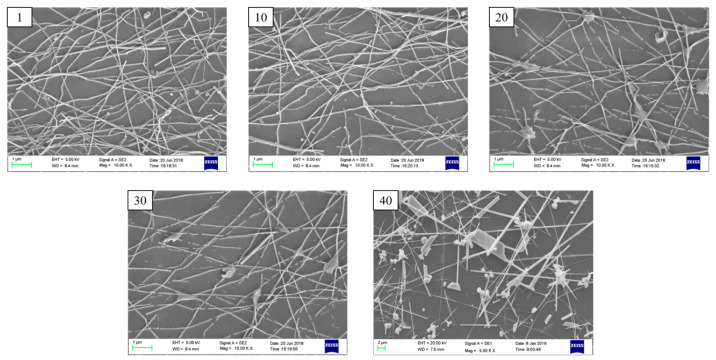
SEM images of the printed AgNWs patterns (2.0 mL silver nanowires ink) with different bending cycles (the numbers in the left upper corner).

It could be seen from the SEM images of the conductive patterns taken at different bending cycles in Figure 8 that when the number of bending cycles reached more than 20, the silver nanowires would be seriously fractured, forming an extremely inferior conductive network of the silver nanowires, which would result in a significant increase in the sheet resistance. However, when the number of bending cycles reached 32, the change of sheet resistance was reduced because the long nanowires were substantially broken into short nanowires, and this degree of deformation was insufficient to cause the silver nanowires to continue to be broken, which led to the conductive network formed by the short nanowires, which tended to be stable. Finally, the sheet resistance remained at around 100 Ω·sq^−1^, and this value still represented good conductivity. Meanwhile, the transmittance of the thin film rose slightly as the number of bending cycles increased. This phenomenon occurred due to the increasing of the sparsity of the silver nanowire network, since the fragmentation of silver nanowires intensified. Owing to the lesser influence on the density of the conductive network, the transmittance changed weakly. According to the characterization results, the printed conductive pattern could still maintain favorable conductivity and transmission after many times of severe deformation. Therefore, it could be determined that the prepared flexible TCFs had excellent stability.

## 4. Conclusions

In this paper, a fabrication of screen printing-based AgNWs flexible transparent conductive film with high stability was reported. The silver nanowires ink was prepared by a self-developed formulation, which was printed onto a flexible transparent substrate, PET, by screen printing. By characterizing the film thickness, sheet resistance and transmittance of the printed pattern, the effects of volume of the ink, temperature and bending times on the film quality were investigated. It was found through the experimental results that the film could still maintain satisfactory sheet resistance and transmittance in the case of multiple severe deformations. It was believed that the encapsulation of the prepared film could further improve its stability. This work was expected to be helpful for the mass production of flexible transparent conductive films and their application in the field of flexible transparent electronic products.
